# My School Teacher

**Published:** 1880-04

**Authors:** J. J. Keyes


### MY SCHOOL TEACHER.

J. J. KEYES.

Let me say, at the beginning, that I know of no such teacher as I shall describe in this article. I do not know that he ever existed. I do not know that he ever will exist. A teacher, like a doctor, or a preacher, may possess many good points, and yet be deficient in certain qualifications that are much to be desired. Were it possible to separate the best points of half a dozen good teachers, and combine them in one person, we should then have the ideal teacher. Several months ago, when the article on "My Doctor" appeared in the BISTOURY, some letters of inquiry were received : "We are chronic invalids; we have tried many doctors, and found no relief. Tell us who *your* doctor is, that we may employ him." But "my doctor" was an ideal doctor, and I

could not name him. Let no School Commissioner, Director, or Trustee inquire for "my teacher." He stands before my mind's eye, distinctly outlined ; a very paragon of pedagogic perfection; but whether he actually presides over any school of boys and girls, I know not. I have never seen him, except "in my mind." Why, then, portray a mere ideality? Simply because ideals are helpful. They stimulate us to broader and higher endeavor. The Jesus of religion helps the race on toward perfection in true manhood. Jesus is the ideal man. Let us, then, have our ideal doctors, preachers, teachers, poets, statesmen, and artists. And if the ideals have never existed, let us form our own ideals, and seek to make them as good and high as possible. School-teaching is very important business, and is second to no other calling. It is a work that bears an intimate relation not only to the intellectual life of the scholar, but also to his forming character ; and is going to determine, bye and bye, very largely, his status as a man and citizen. The influence of school life is an every day influence ; and every day influences are those which tell most largely on the character. School teaching, therefore, demands the highest order of gifts and qualifications, as truly as law, medicine, or the Christian Ministry. Of course, then, my school teacher is an *educated* man. This should go without saying. His work is an educational work. Therefore, he must needs be educated. He must, at least, be fitted by education, for the grade of school in which he is to teach. He does not absolutely need the same degree of mental culture for a district school, that he would for a University professorship, though I am not sure that, even as a country school teacher, he would not be a much greater success, other things being equal, if he were college bred. I think he would. As a rule, however, such talent commands a price which the country school district cannot pay. All that I insist upon, therefore, is that my school teacher shall be amply qualified, so far as he can be by education, for the position he is to occupy. School children are not slow to detect the ignoramus. The ignoramus will not answer. The Latin professor in a certain seminary I wot of, who did not understand the construction and grammar of the language as well as half his pupils would not answer, and was obliged to "step down and out." My school teacher is fully educated, up to the point required by his position.

My school teacher is, also, "*apt to teach.*" He has the teaching gift—a genius for the work. "*Poeta nascitur, not fit.*" The same is true, in a measure, of the ideal teacher. He is a *born* teacher. He has a native aptitude for communicating what he knows. He has the faculty of imparting and explaining. The gift may be enlarged by use and cultivation ; but, first of all, it is a gift. It is a certain something that specially fits a person for the work of teaching. To what extent it may be developed in one who seems to lack it, I do not stop to ask. The teacher who lacks it, as an endowment, however much he may develop and cultivate it, can never become my ideal teacher. A certain gracefulness, naturalness, and ardor will be wanting. There will be something stiff and mechanical in his methods and manner, in the school room. My school teacher was "to the manner born," and has unmistakable aptitudes for his work.

I wish to say, somewhere in this article, that my school teacher is a good specimen of *manhood.* I might as well say it here. He is not simply a man of knowledge. He is a man of character, who commands the respect of his scholars for what he is, as well as for what he knows. He is a man of large ideas of life, and of things manly and honorable. He never descends to acts of petty meanness and tyranny in his treatment of those who are placed under his charge. Parents, little by little, reach the gratifying conclusion that their children will suffer no injustice at his hands, because they find that he himself is a just man, and a fine specimen, every way, of true manhood. And when he mingles in society, or walks the streets, people do not say : "There goes that contemptible, narrow-minded, beastly school teacher of ours;" but, "There goes one of the manliest fellows and best men of this town." He enjoys the respect and esteem of the whole community. His pupils, also, regard him with mingled reverence and affection. His manly character continually excites their admiration.

Furthermore, my school teacher *loves to teach.* He does not teach for a living. He does not teach for his salary, though the salary comes handy. He does not teach because he must *do* something, and because he might as well teach as do anything else. Neither does he teach because he is too proud to work at farming, or a trade. He teaches, not from mercenary motives, or motives of pride, or motives of convenience, nor as a temporary makeshift; but because he loves teaching, and because he feels himself called to it and adapted to it. Many a young man and young woman, when in pursuit of an education, having been asked what they intend to do when their school days are over, have said: "We hardly know; but if we can't do anything else, we can teach;" when, perhaps, teaching is the last thing in the world for which they have the needed qualifications. He who has no real love for the work, and does not feel himself drawn to it, would better leave it alone, no matter how highly educated he may be. It is to be feared that many are to-day engaged in this calling, who would make very good clerks, mechanics, servants, dress makers and milliners, who succeed only indifferently well as teachers, because they have taken up the work as a matter of convenience and respectability. They are not in love with it. In the morning, they enter the school room with reluctant steps, and say to themselves: "O, this weary day! how am I to live through it!" And at night: "Thank goodness, one more day is gone." And the scholars are even more thankful than they. My school teacher loves to teach. He enters the school room, each day, eager for his work, rejoicing to see the budding and blossoming processes that are going on under his hands, in love with his pupils, and glad to be of service in fashioning them to a beautiful womanhood and a noble manhood. And, although he may often weary in the service, he never wearies *of* the service; for he loves it; it is his meat and drink: and he has willingly made it his life-work.

My school teacher is, also, a very patient, hopeful, cheerful man. It is not always easy for him to be such, but he schools himself to it, and will not consciously allow himself to be otherwise. He has many things to try him. His scholars are of all sorts, from all classes of society. Their home training has been various. The influences surrounding them out of school, have been good, bad, or indifferent. Many have not been educated to any self-restraint, nor to habits of application; and, in the school room, they are thoughtless, listless, disrespectful to their teachers, quarrelsome, mischievous, and impatient of all authority. My school teacher looks upon them more in pity than in anger. He considers that they are only children, lacking judgment, spoiled in temper, ignorant of the world, and unaware of the responsibilities, struggles and sorrows that await them as grown-up men and women. But he knows them to be possessed of rational minds. He sees in them possibilities of life, character and intelligence. And so, with great patience, hopefulness, and good nature he addresses himself to his task. The primary sense of patience is "continuance, holding out." It implies the calm endurance of a steady strain. A teacher, who is responsible for the guidance and government of a school, is never free from this strain. From the moment he enters the school room until he leaves it, his powers are all in a state of tension. He is occupied with details, and is continually concerned for the orderly, harmonious movement of school affairs. He is like a general, who has marshaled his forces, and gone into an engagement. A general needs patience, needs equilibrium, needs to "keep his head;" for he has a great deal to see to; he must direct all movements, and assume all responsibilities; and the strain is often fearful. So with my teacher. With all his planning and organizing, and with the best of lieutenants, the school will not run itself. He must have an eye to everything. The general movement of things is under his direction, and he must have an unfailing patience.

Hopefulness is a characteristic of equal prominence. My school teacher never despairs of results. He never despairs of the stupidest, or most unruly scholar in the school. He never despairs of bringing about an era of perfect order and pleasing prosperity. His hopefulness, being combined with enthusiasm, becomes at length an inspiration to his pupils. If *he* believes in them, why should they not believe in themselves? If he is hopeful in their behalf, why should they not be hopeful on their own account? He communicates his spirit to them. His unfailing hopefulness creates an atmosphere in which his pupils, during school hours, "live and move and have their being." His enthusiasm becomes contagious; they all catch it, sooner or later. But whether all catch it or not, he perseveres in it, and never allows himself to be altogether discouraged. He does not command them to willing obedience and faithful study, but incites them to it, by his out-breathing hopefulness and unflagging enthusiasm. Cheerfulness follows, as a matter of course. He does not move around in his little kingdom, and among his juvenile subjects, with a clouded brow. He does not go to the school room in the morning with an aching head and a bearish temper, produced by a previous night of sleeplessness, or dissipation, or pleasure seeking at fashionable parties; and then inflict a part of the penalty upon the unoffending children. He does not come before his class with an air of tired indifference, nor repel the scholar, who seeks information, with a "Get away with you to your seat. I can't be bothered now." He does not fret, and fume, and storm at little mistakes, or slight disorders, nor petulantly declare the scholars to be a pack of rebels and numskulls. He does not seek to annihilate the dull boy or girl with ridicule, sarcasm, and biting, cruel satire, nor try to bring upon them the laugh of the whole school. No, no: my school teacher is a gentleman: he looks upon his scholars as human beings, made in God's image, and entitled to some consideration: and so he wears a cheerful countenance, excites their hopefulness, and bears with their foibles and stupidity with great patience. All this requires great self-control. The strain is constant and sometimes severe; but he nerves himself to it; and the hourly cry of his soul is, "*Nil desperandum.*" God bless him. He is a hero of the noblest type.

My school teacher, I will say further, so teaches and trains his pupils as to make independent thinkers and investigators of them. He is not merely a man of the book. He is not slavishly confined to "the author." He does not limit himself to the prosaic statement: "The book says." Neither is he content to have his pupils cram their minds with the simple contents of their text books. Indeed he does not believe in the cramming process at all. When stupid Boards of Education prepare a schedule, and say to him: "Here, sir, follow that; and see to it that your scholars compass it in the course of the year, and pass with credit all the Regent's examinations," he groans in spirit, commiserates his scholars, and anathematizes the board of education. No, my school teacher doesn't like that. He has a higher conception, both of education, and also of school teaching, than belongs to such a system. To his notion, text books are means, not ends. They are instruments, not the finished work. Their contents are not simply to be mastered by the memory. The mind is not to be stored from top to bottom with dry facts and abstract knowledge. The true philosophy of education is not understood by those who would have the student commit so many rules of grammar, or arithmetic, or so many pages of history, geography or physiology each day, and recite the same with due accuracy and regularity at the recitation hour. Education is not the mere imparting of knowledge by the teacher, nor the mere acquisition of knowledge, as contained in books, by the scholar; and when the scholar is through with school, he should come out, something more than a well trained parrot, or mocking bird. It is the province of the teacher to excite the inquisitiveness of his pupils, set them to looking after the reasons of things, and train them to independent thinking. The teacher who is not suggestive and inspirational, and does not succeed in getting his pupils beyond rules, formulas and dry facts, to a knowledge of principles and a passion for inquiry, falls most wofully short of the mark.

My school teacher also believes that more prominence should be given in our primary schools to studies in physical science, and in matters of practical importance, pertaining to every day life. It is the few, only, who go from the primary to the academic, and from the academic to the collegiate ranges of study, and thus become versed in physical science, and in a practical philosophy of life. And I think (or rather, my school teacher thinks) it is an open question, whether we are doing all that can and should be done for those whose education begins and ends at the primary schools. Prof. Huxley says, with great truth and force: "At present, education is almost entirely devoted to the cultivation of the power of expression, and of the sense of literary beauty. The matter of having anything to say beyond a hash of other people's opinions, or of possessing any criterion of beauty, so that we may distinguish between the god-like and the devilish, is left aside as of no moment. I think I do not err in saying that if science were made the foundation of education, instead of being, at most, stuck on as a cornice to the edifice, this state of things could not exist." And again: "One is constantly asked, when should this scientific education be commenced? I should say, with the dawn of intelligence. A child seeks for information about matters of physical science as soon as it begins to talk. The first teaching it wants is an object lesson, of one sort or another; and as soon as it is fit for systematic instruction of any kind, it is fit for a modicum of science." Is it not a fact that when, at the age of sixteen or seventeen years, the school life of the larger number of our boys and girls shall have ended, they will be confronted, at almost every step, in any calling of life that may be chosen, with facts, problems, and questions, which a knowledge of physical science in its various branches, would enable them to understand and profit by? And do we not overlook their necessities and do them a wrong, by confining them too long to the orthodox (?) curriculum of our public schools, and refusing them the knowledge of a science that would be of such real value to them for many of the practical uses of life? My school teacher thinks so, and says that the true education of our children is that which will best fit them to become well informed, self-poised, efficient men and women, in the spheres of life which they are destined to occupy. I cannot give his views on this point any further, in this article, though I would like to; for he has one other peculiarity to which I must call attention, and I must hasten on. That peculiarity is this: His school government is a scheme for the *development of character*. Unlike many teachers, he does not resort, very frequently, to repressive measures and arbitrary prohibitions. The ferule, raw-hide, and hickory switch are not found in his school-room. Corporeal punishment is no part of the discipline, with him. Little New Jersey manages to do without it; and he thinks the schools of other states, and of all the states, and of all the world, would be better off without it. Just here, he asssumes to differ from Solomon, and believes that he who spares the rod *loves* the child, and can produce, by other means, far better results of behavior and character. And so he never uses either the rod, or any kind of physical torture, to compel a scholar to good behavior and studious habits. Indeed, he has few, if any, ironclad, stringent rules. On the contrary, he allows liberties which many teachers think are dangerous to the good order of the school. And yet, strange to say, his school seldom presents a scene of confusion, and is, usually, quiet in the extreme. If an abuse of liberty occurs, he reasons kindly with the offendor, points out his wrong, shows him the injury such a habit will work to the good order of the school, and what a damage it will work in his own character. He believes that all children, even the worst, have, somewhere within them, a vein of reason, a spark of conscience, and a latent sense of honor. He aims to develop these, by timely appeals, by frequent illustrations of their beauty and excellence, and by drawing vivid contrasts between conduct that springs from reason, conscience and a sense of honor, and conduct that springs from willfulness, selfishness, ignorance, and a love of evil. He believes it to be a part of his work to make of those boys and girls, true men and true women; and to do this, he not only shows them the things that are right; he also trains them to habits of thoughtfulness, self-examination and self-correction. He puts them largely in their own keeping, giving them right principles to guide them. When they slip, or transgress, he points out the difficulty, and renews his advice. He appeals constantly to their reason, sense of right, and sense of honor. And he never allows himself to be discouraged. He keeps doing it, DOING IT, DOING IT; and is entirely satisfied with the results. Thus he works away, laying deep and broad the foundations on which our children may build noble, beautiful, enduring characters, and come forth from school, by and by, armed and equipped for the actual battles of life. Without note or comment, or further description, we will bid my school teacher good day.

